# Supplementary material for: Dysbiosis in Human Urinary Microbiota May Differentiate Patients with a Bladder Cancer
Source: Int J Mol Sci. 2024 Sep 21;25(18):10159. doi: 10.3390/ijms251810159 (PMC11432408; doi:10.3390/ijms251810159)
Supplement: Supplementary file 1 [file ijms-25-10159-s001.zip › Figure S1.pdf]

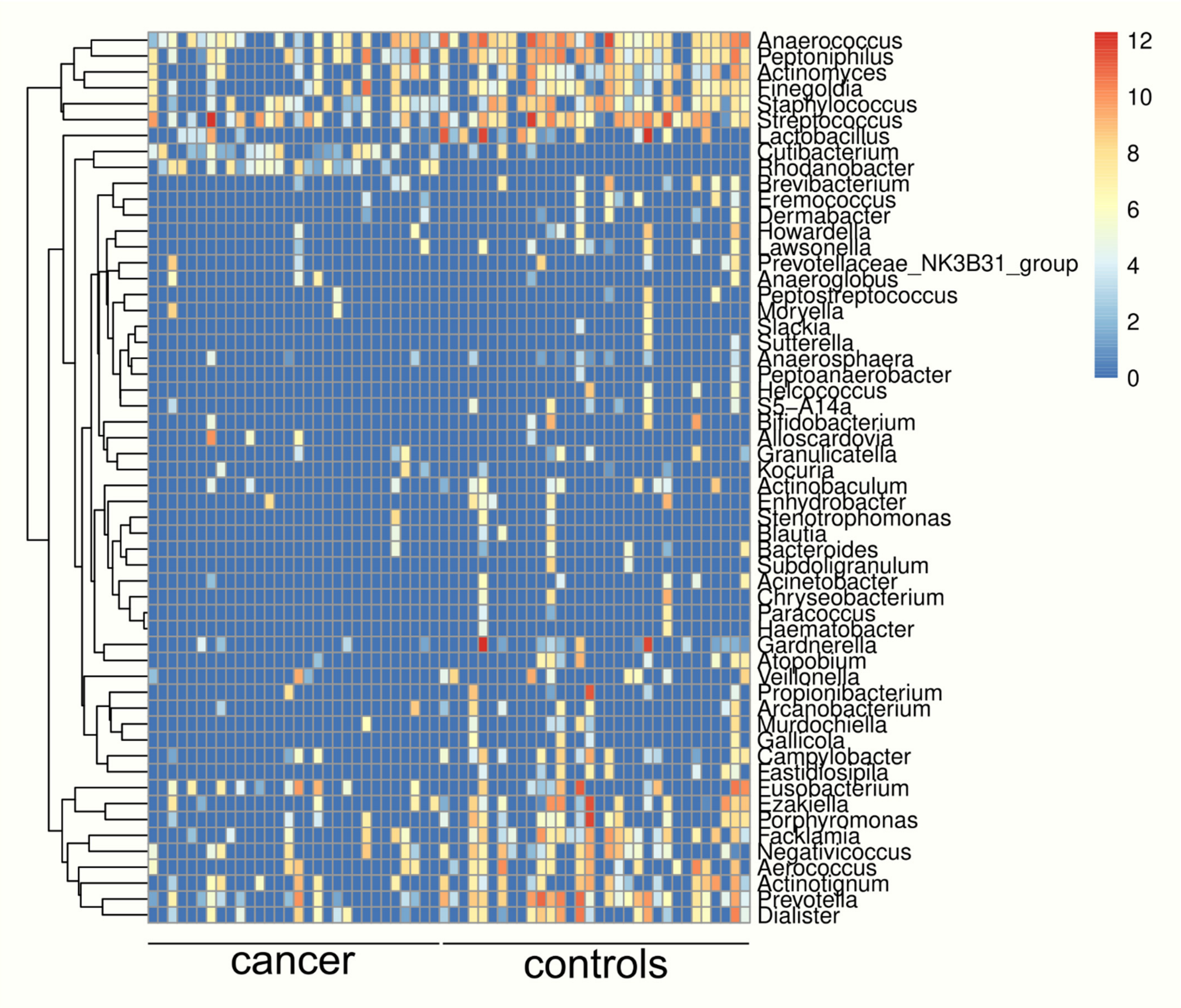

Figure S1. Heatmap representation of the relative abundance of genera across urine samples from bladder cancer and healthy volunteer samples. Each column represents an individual sample and each row correspond to a genus. The color intensity reflects the normalized relative abundance of each genus within the sample.
